# Improving emergency obstetric care and reversing the underutilisation of vacuum extraction: a qualitative study of implementation in Tete Province, Mozambique

**DOI:** 10.1186/s12884-018-1901-3

**Published:** 2018-06-27

**Authors:** D. Geelhoed, V. de Deus, M. Sitoe, O. Matsinhe, M. I. Lampião Cardoso, C. V. Manjate, P. I. Pinto Matsena, C. Mosse Lazaro

**Affiliations:** 1 Tete Provincial Health Directorate, Rua de Macondes, Cidade de Tete, Tete Province Mozambique; 2 Tete Provincial Hospital, Tete Provincial Health Directorate, Rua de Macondes, Cidade de Tete, Tete Province Mozambique; 3 Rural Hospital of Mutarara, Tete Provincial Health Directorate, Rua de Macondes, Cidade de Tete, Tete Province Mozambique; 4 Rural Hospital of Ulongue, Tete Provincial Health Directorate, Rua de Macondes, Cidade de Tete, Tete Province Mozambique; 5 District Services of Health, Women and Social Action of Chifunde, Tete Provincial Health Directorate, Rua de Macondes, Cidade de Tete, Tete Province Mozambique; 6 District Services of Health, Women and Social Action of Cidade de Tete, Tete Provincial Health Directorate, Rua de Macondes, Cidade de Tete, Tete Province Mozambique

**Keywords:** Emergency obstetric care, Vacuum extraction, Maternal mortality, Stillbirth, Mozambique

## Abstract

**Background:**

Maternal and perinatal mortality in Mozambique were declining at a slow pace, despite progress in coverage of institutional childbirth. Implementation of quality emergency obstetric care including vacuum extraction remained inadequate. In 2015–2017, Tete Province achieved remarkable progress in improving emergency obstetric care and reversing the underutilisation of vacuum extraction, with encouraging results for maternal and perinatal outcomes, despite severe resource constraints. This paper presents the experience of Tete Province, generating a rich, contextualised understanding, which might provide generalizable insights and lessons.

**Methods:**

This qualitative study design is used to present Tete’s experience in improving emergency obstetric care and reversing the underutilisation of vacuum extraction, drawing on principles from implementation science and applying a systems thinking approach. Sources include routine data, documents, social media messages, and the lived experience of the authors, all intimately involved in the implementation process during 2014–2017. Iterative learning and analysis, involving all authors, led to the final interpretations.

**Results:**

Within a context of severe resource constraints, Tete applied 4 interventions (training, accreditation, audit, monitoring and evaluation with feedback) to improve the implementation of emergency obstetric care. Considerable progress was achieved in vacuum extraction and other signal functions of emergency obstetric care and in the decision-making process for caesarean sections, contributing to important reductions in the provincial institutional maternal mortality and stillbirth rates. Facilitating factors include attributes of the vacuum extraction itself, of the structural and organisational environments in which it was introduced, of the people involved in implementation, and of the process through which the implementation was rolled-out.

**Conclusions:**

The lessons from implementation science and systems thinking can contribute to surprising results in the improvement of emergency obstetric care including the use of vacuum extraction, even in a severely resource-constrained setting. The creation of conditions for real change, with empowerment of the staff and managers at the front-line of day-to-day practice in Tete may inspire others in similar conditions and circumstances. The underutilisation of vacuum extraction in middle- and low-income countries is indeed a missed opportunity. Its reversion is possible and provides a good chance to make considerable difference in maternal and perinatal outcomes.

## Background

Mozambique is one of many countries which have not managed to reduce their maternal mortality fast enough to achieve Millennium Development Goal 5 (reduction of maternal mortality ratio by three quarters between 1990 and 2015), despite increasing access to, and utilisation of, institutional childbirth. Similarly, the country’s progress in the reduction of under-five mortality has not been fast enough to achieve Millennium Development Goal 4 (reduction of under-five mortality rate by two thirds between 1990 and 2015), mostly because neonatal mortality has not reduced much [1, 2]. It has been suggested that an adequate coverage of skilled birth attendants is not sufficient to improve maternal and perinatal outcomes, but that good coverage and quality of emergency obstetric care are additional prerequisites [3–9]. Of the essential emergency obstetric interventions, vacuum extraction is most underutilised in low and middle income countries [10–15]. Instead, there is often an excessive dependence upon caesarean section [16], despite its limited access in many low-income countries, such as Mozambique. Aiming to accelerate progress within the framework of the Sustainable Development Goals, improved access and quality of maternity care form an important objective in the current strategic plans of the Mozambican Government and its Ministry of Health.

Mozambique has had clear recommendations for emergency obstetric care for many years, based on WHO recommended practices. These include nine signal interventions: administration of antibiotics, oxytocin, magnesium sulphate and blood transfusion as well as performance of caesarean section, vacuum extraction, manual removal of placenta, neonatal resuscitation, and intra-uterine aspiration [17, 18]. In addition, the country implements a successful task-sharing strategy for the provision of emergency obstetric care and midwives are trained in the use of vacuum extraction [19]. However, widespread implementation of all essential emergency obstetric interventions remains a challenge, and both underuse and overuse of caesarean sections has been identified [20]. As elsewhere in Mozambique and similar countries, Tete has been trying to improve obstetric emergency care for years, especially care for prolonged labour, which was locally identified as an important cause of institutional maternal mortality and stillbirth [21]. Despite these efforts, according to routine data in the last quarter of 2014, only 8 of Tete’s 106 health facilities performed all signal interventions for basic or comprehensive emergency obstetric care, indicating very limited access to emergency obstetric care for the 2.5 million inhabitants living dispersed in the mostly rural province. Vacuum extraction was the least performed signal function, few direct obstetric complications were reported and case fatality rates for each of the five direct obstetric complications were above 1%.

In 2015–2017, after implementation of a series of mostly non-clinical interventions, Tete Province managed to achieve remarkable progress in improving emergency obstetric care and reversing the underutilisation of vacuum extraction, with encouraging results for maternal and perinatal outcomes, despite severe resource constraints. Few such successful experiences in low-income countries, especially from Sub-Saharan Africa, have been documented, rarely addressing the underutilisation of vacuum extraction [22–28]. This paper aims to comprehensively present the experience of Tete Province to generate a rich, contextualised understanding, providing generalizable insights and lessons for replication.

## Methods

This paper uses a qualitative study design to present Tete’s experience with improving emergency obstetric care and reversing underutilisation of vacuum extraction, drawing on principles from implementation science and applying a systems-thinking approach. Implementation science studies the phenomena related to implementation of evidence-based health innovations in complex real-life health-related settings [29, 30]. Adam & de Savigny (2012) described systems thinking as a way of thinking in approaching problems and designing solutions, which frames problems in terms of patterns of behaviour over time and places the responsibility for this behaviour on the system’s internal actors. For a deeper understanding of the actors’ behaviour it is considered crucial to understand their relationship context, while concentrating on causality, understanding how the behaviour is generated and maintained, viewing causality as an on-going process, with effects feeding back to influence causes and the causes affecting each other [31].

This approach enables not only a description of implemented interventions and achieved results in Tete Province, but more importantly, of how these interventions and results were implemented, achieved and maintained, aiming to provide useful clues for replication elsewhere. The authors were all intimately involved in this experience and its evaluation during their day-to-day work before, during and after 2015–2016, at several levels and with different perspectives, including health system and program management (province, district, facility), medical-technical clinical care, and midwifery. Their combined empirical knowledge ensures a profound understanding of the implementation process, achieved results, and the context in which these occurred. In addition, the study draws on routine data from the provincial health information system, routine progress reports from the provincial health sector, reports on quarterly accreditation in emergency obstetric care, minutes from meetings of the provincial committee for maternal and neonatal death reviews and clinical audits. It also used general non-identifiable information related to emergency obstetric care extracted from the routine managerial communications within the provincial health sector via pre-existing WhatsApp Messenger groups (an instant messaging service for smartphones from WhatsApp Inc., wholly owned by Facebook) between the Provincial Health Directorate and its district health directors, district medical officers, and other relevant managers. Emerging interpretations were discussed and agreed between the authors, as well as consolidated with a wider audience of district managers and health care professionals in the province. The use of existing data for this study from all sources mentioned above has been authorised by the ethics committee of the Faculty of Health Sciences of the Zambeze University in Tete.

## Results

### Context

Tete province is situated in the central region of Mozambique in south-east Sub-Saharan Africa, one of the least developed countries in the world [32]. The province has a surface area of 100,724 km^2^ subdivided in 15 districts, a population density of around 25 inhabitants per km^2^ and a largely underdeveloped transport infrastructure. In recent years Tete has experienced a mineral resources boom, resulting in important socio-economic changes and considerable population growth due to national and international immigration. Despite this, the local economy is still mostly based in subsistence agriculture and poverty levels and inequality are high [33]. Literacy levels are low, especially among women, and access to safe water and sanitation or improved housing limited [34]. The epidemiological profile of the province identifies a multiple burden of diseases, with persisting high levels of malaria, diarrheal diseases, HIV/AIDS and other infections, frequent occurrence of health problems related to reproduction and emerging problems of non-communicable diseases and trauma [35]. A description of the provincial health sector is presented in Table 1.

**Table 1:** Health sector profile in Tete Province, Mozambique

	Situation in Tete Province (2014–2015)	Targets (Mozambique)
Institutions	• Provincial Health Directorate, 15 subordinate district health services;	• 1 district hospital with surgical capacity per district (total 15);
	• 1 provincial hospital, 3 rural hospitals, 1 district hospital, 120 primary care facilities;	• > 250 health facilities (1 per 10,000 inhabitants), in a catchment area with a radius of < 10 km;
	• 6 health facilities with surgical capacity (1 since end 2015);	
	• on average, 1 health facility per around 20,000 inhabitants in a catchment area with a radius of approximately 16 km;	
Health personnel	• 75.9 per 100,000 population, of which 35.2 medical doctors, general nurses and Mother and Child Health (MCH) nurses (of which 14.7 MCH nurses);	• > 113 per 100,000 population, of which > 77 medical doctors, general nurses and MCH nurses;
	• 5 specialist obstetricians concentrated in the provincial hospital;	• specialist obstetricians in provincial and all rural hospitals.
	• 1 general medical doctor for supervision and support of health care in most districts.	
Maternity care	• nearly all health facilities provide MCH-nurse-led maternity services	• all health facilities provide MCH-nurse-led maternity services;
	• all health facilities attending at least 100 institutional childbirths per 3 months, are included in the quarterly emergency obstetric care accreditation process;	• all health facilities attending at least 100 institutional childbirths per 3 months, gain accreditation in emergency obstetric care in each quarterly evaluation;
	• caesarean sections and other obstetric surgeries are usually performed by surgically trained health technicians, some with a background as MCH nurse.	• WHO recommends at least 5 facilities accredited in basic emergency obstetric care and 1 facility accredited in comprehensive emergency obstetric care per 500,000 population, or more if distances are great, which translates to a minimum of 25 and 5 accredited facilities, respectively, in Tete Province.
	• Tete provincial hospital: 320-bed tertiary facility with large maternity ward accredited as ‘model maternity’ in 2014; usually overloaded with uncomplicated births, leaving little capacity for more complicated cases and referrals; insufficient theatre capacity, regularly causing delays to surgery;	
	• a private health care sector is practically absent, with just one private clinic licensed to attend childbirth, without capacity for emergency obstetric care.	

The Mozambican public health sector is financed by the government. In 2014, it was allocated MT 19.1 billion (USD $635.8 million), 7.9% of the total state budget, equivalent to approximately USD $42 per person, far below the SADC average of USD $266 [36]. However, the sector also receives important financial contributions (estimated at an additional 25%) from a multitude of development partners, and is still considered donor-dependent [36]. Tete provincial health sector receives considerable support from bilateral and multilateral donors, and from national and international non-governmental organisations. An important part of this support is directed at HIV/AIDS (largely funded by the Government of the United States of America), while another part focusses on health system strengthening to improve sexual and reproductive health and rights, within which attention has been directed at the improvement of emergency obstetric care (largely funded by the Government of Denmark). In Mozambique, maternity care is free of charge at point of care, as are most other health services for vulnerable population groups.

### Interventions

Improvement of maternal and perinatal outcomes had been considered a priority in Tete province for years, following the lead of the Mozambican Ministry of Health. Till 2014 most efforts were directed at promotion of institutional childbirth (through expansion and refurbishment of maternity services and health education) and of modern contraception (through health education and the introduction of new methods of long duration, including implants), with considerable success: institutional childbirth coverage increased from around 50% in 2010 to over 66% in 2014, and the number of couple-years protected per 100 women of reproductive age increased from close to 13 in 2011 to over 32 in 2014. Despite this progress, the provincial institutional maternal mortality rate and stillbirth rate remained high at 100–150 per 100,000 births and around 18 per 1000 newborns, respectively. Provincial managers recognised that coverage and quality of maternity care including emergency obstetric care was insufficient and required interventions. In Mozambique, vacuum extraction tended to be considered as a rather risky practice with many potential dangers for mother and child, and was practiced in only a few health facilities. The practice of craniotomy (perforation of the head of a dead foetus to evacuate its content followed by extraction of the foetus) was even less known about, and considered potentially culturally unacceptable. Despite this, Tete’s provincial health management team authorised implementation of four complementary interventions to address the situation from March 2015 onwards: 1) one-week training of a provincial pilot team on the management of prolonged labour, including vacuum extraction and craniotomy, developed and facilitated by a professor considered an expert on obstetric care in low-income countries both internationally and in Mozambique; 2) strengthening of the quarterly emergency obstetric care accreditation process; 3) monthly audit of clinical files of all cases of caesarean section in the provincial hospital (a large majority of all caesarean sections in the province) with specific feedback to clinicians involved, copied to relevant provincial managers; and 4) strict monitoring and evaluation of routine data from all maternities, with specific feedback to district health directors and doctors, copied to all and to relevant provincial managers. These four non-clinical instruments (training, monitoring and evaluation, audit and constructive feedback) have been shown effective in changing behaviour of health professionals [23, 37–42]. They are presented in more detail in Table 2.

**Table 2:** Interventions

Intervention	Description
Training of provincial pilot team	• recommended use of vacuum extraction by MCH nurses, doctors or obstetricians to shorten the second phase of labour for maternal or foetal benefits, allowing a maximum of three tractions to achieve childbirth, with referral and caesarean section as alternative. Its use was based on four criteria: 1) complete dilatation of the cervix; 2) cephalic presentation; 3) gestational age at term; 4) descent of the presenting part at the third or fourth planes of Hodge;
	• coincided with the introduction of hand-held devices for vacuum extractions (Kiwi™ Omnicup, sterilisable version), easy to use although with little resistance to regular re-use; an additional number of vacuum extraction cups (both metal and silicon) and pumps (hand-operated, foot-operated or electrical) were already available;
	• staff were encouraged to experiment with the assembly of a functional vacuum extractor with any available types of cup and pump and to practice regularly (at least once a month) to gain experience and promote emergency preparedness;
	• no other staff received additional formal training to perform vacuum extraction, but clinicians at the provincial hospital were assigned to provide in-service training to any MCH nurse or doctor wishing to strengthen their capabilities;
	• sharing of capabilities between staff at district or health facility level was actively encouraged and regularly occurred (as shown by photos and comments in the relevant WhatsApp groups), with or without additional facilitation from provincial level.
Monitoring and evaluation	• the quarterly emergency obstetric care accreditation process was based exclusively on routine data in the provincial health information system;
	• each quarter, data from all participating health facilities were compiled in a spreadsheet with numeric and graphic representations of performance, focussing on vacuum extractions and health facilities accredited in emergency obstetric care; these results were included in the routinely prepared quarterly performance reports of the provincial health sector.
Audit	• a monthly audit of clinical files of all cases of caesarean section in the provincial hospital was performed from January 2015 till September 2016 (and once more for March 2017) by a locally-based international technical advisor, not directly involved in patient care;
	• focussed on the appropriateness of the indication for caesarean section and previous use of less invasive methods to accelerate labour;
	• a caesarean section was considered unavoidable in case of: lack of progress after artificial rupture of membranes and augmentation with oxytocin, repeated failed induction of labour with misoprostol, failed attempt at vacuum extraction, two or more previous caesarean sections, placental abruption, non-cephalic presentation with present foetal heartbeat, ruptured uterus, placenta praevia, or cord prolapse with present foetal heartbeat, as documented in the clinical files;
Feedback	• quarterly feedback on the accreditation in emergency obstetric care was provided via email and Whatsapp groups to provincial managers, district directors, district medical officers, and hospital obstetric staff, after a previous reminder to pay extra attention to health facilities which were close to accreditation based on data from the first two months of each quarter;
	• monthly feedback of the audit results was provided to clinicians involved included the percentage of potentially avoidable caesarean sections per clinician, visible to the whole team.
	• well performing MCH nurses, doctors, health facilities and districts were praised in the relevant WhatsApp groups, while others were encouraged to follow their example and try harder;
	• in 2016, the staff of one health facility and the corresponding district health director and doctor which managed to achieve accreditation in six consecutive quarters, as only health facility in the entire province, received public recognition and a prize in the principal annual provincial health sector meeting; several districts also organized prize- and recognition ceremonies for well performing staff and health facilities at local level;
	• due to staff changes at provincial level affecting the chief medical officer, chief public health officer, MCH manager and locally-based international technical advisor, the accreditation process received less attention in 2017 and specific feedback was not provided.

The focus on vacuum extraction was chosen because it is a relatively safe intervention even in inexperienced hands, with ample scientific evidence for its potential to reduce maternal and perinatal morbidity and mortality [5, 10, 13, 28]. Craniotomy and other destructive procedures have also long been considered safer than caesarean section in cases of prolonged and infected labour with intra-uterine foetal death [43, 44]. As no specific budget had been allocated to these interventions during the annual planning process for 2015, all interventions occurred within routine activities and without additional financial incentives, except the training, which was funded through the program for health system strengthening to improve sexual and reproductive health and rights.

### Progress achieved

**Fig. 1:**
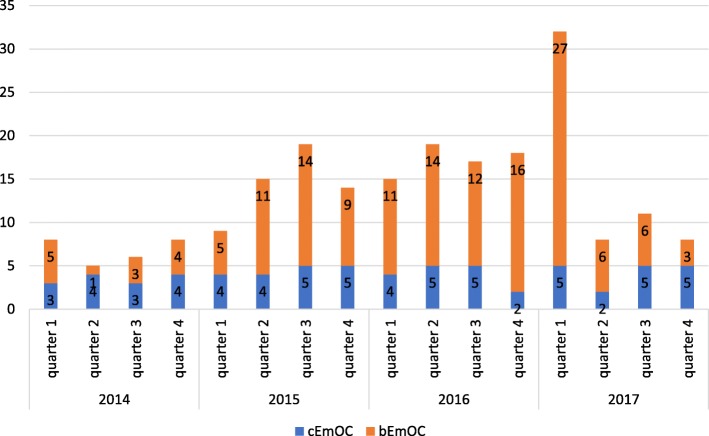
Evolution of the number of health facilities accredited in basic and comprehensive emergency obstetric care

The number of health facilities quarterly accredited in basic or comprehensive emergency obstetric care is presented in Fig. 1. After mid 2015 vacuum extraction was no longer the least performed signal intervention, replaced in that position by intra-uterine aspiration, administration of antibiotics or of magnesium sulphate. The performance of all signal interventions improved, as any one was performed each quarter on average by 25 health facilities in early 2015, increasing to 44 at the end of 2016, and to 47 in the first quarter of 2017, after which it reduced somewhat and stabilised around 42 during the remainder of that year. As a result, considerable changes were observed in the coverage of emergency obstetric care at provincial level, although some progress was lost during 2017 (Table 3). The use of vacuum extraction increased considerably from 2014 to 2016, and stabilised during 2017, while the percentage of institutional births assisted by caesarean section reduced (Table 4). Not only the absolute number and the percentage of institutional births assisted with vacuum extraction increased dramatically, but also the number and proportion of health facilities which performed at least one vacuum extraction during each three-month period. The percentage of institutional births assisted by either vacuum extraction or caesarean section therefore increased considerably, especially in district and peripheral health facilities without surgical capacity.

**Table 3:** Changes in provincial coverage of emergency obstetric care, 2014–2017

	2014	2015	2016	2017	Evolution 2014–2017
Provincial coverage of institutional births (as percentage of expected births)	66.5%	71.0%	75.8%	76.6%	15.2%
Percentage of institutional births attended in accredited health facilities (province)	19.9%	33.8%	31.8%	24.8%	24.6%
Percentage of expected births attended in accredited health facilities (province)	13.3%	23.1%	23.5%	19.0%	42.9%

**Table 4:** Changes in the use of vacuum extraction and caesarean section at provincial level, 2014–2017

	2014	2015	2016	2017	Evolution 2014–2017
Number of vacuum extractions	176	824	2034	2073	1078%
Number (proportion) of health facilities which, on average, perform at least one vacuum extraction per quarter	14 (13%)	21 (18%)	45 (36%)	44 (33%)	214%
Percentage of institutional births assisted by vacuum extraction	0.2%	1.0%	2.3%	2.2%	1000%
Percentage of institutional births assisted by caesarean section	2.7%	2.1%	2.1%	1.8%	−33%

The monthly audit of the clinical files of all caesarean sections in the provincial hospital revealed that, initially, surgery occurred often ‘too little, too late’ but also ‘too much, too soon’ [45]. Similar audit findings have been described for various hospitals in Tanzania [46–48]. After interventions started in April 2015 changes occurred gradually, accelerating during 2016, as shown through selected indicators in Table 5. The observed reduction of births attended in the hospital appeared to be related to the inauguration of an additional Mother and Child Health (MCH) nurse led maternity close by and to an increased use of peripheral maternities in case of uncomplicated births. Craniotomy and other destructive procedures were introduced as a substitute to caesarean sections in cases of intra-uterine foetal death, without any objections of patients or their families after appropriate explanations with informed consent. The persistently high intra-hospital caesarean section rate was probably related to the changing case-mix of relatively more severely complicated births and referrals, as also suggested by the increasing concentration of maternal deaths in the provincial hospital. It appeared that the shift in indications for caesarean section had reduced at least part of those ‘too little, too late’ as well as of those ‘too much, too soon’, without compromising maternal and foetal outcomes.

**Table 5:** Changes in selected indicators in Tete Provincial Hospital, 2015–2016

	Jan-Mar 2015	Apr-Sep 2015	Oct 2015-Mar 2016	Apr-Sep 2016	Evolution 2015–2016
Number (percentage) of institutional births in the province which occurred in the provincial hospital	1400 (8.4%)	2630 (6.6%)	2455 (5.8%)	2199 (3.9%)	(−53.6%)
Intra-hospital caesarean section rate	23.1%	24.2%	25.2%	29.1%	26.0%
Percentage of caesarean sections considered inevitable^a^	23.5%	29.2%	28.6%	35.2%	49.8%
Percentage of caesarean sections considered inevitable after referral from periphery^a^	23.8%	32.8%	30.7%	35.8%	50.4%
Percentage of caesarean sections with intra-uterine foetal death (percentage considered inevitable^a^)	10.2% (44.8%)	8.2% (64.4%)	8.0% (68.2%)	12.1% (71.4%)	18.6% (59.4%)
Number of readmissions after caesarean section for wound dehiscence (percentage of caesarean sections performed)	13 (4.0%)	4 (0.6%)	4 (0.6%)	6 (0.9%)	(−77.5%)
Percentage of caesarean sections on nulliparous women with a single, term foetus in cephalic presentation (Robson classification 1)	41.8%	39.1%	32.7%	32.2%	−23.0%
Percentage of repeat caesarean sections (Robson classification 5)	25.3%	23.7%	28.4%	28.3%	11.9%
Percentage of asphyxiated newborns after caesarean section (Apgar score at 5 min < 7)	9.4%	5.8%	9.4%	8.1%	−13.8%
Intra-hospital maternal mortality rate per 100,000 intra-hospital childbirths (proportion of total provincial maternal deaths)	71 (8.3%)	456 (57.1%)	937 (71.9%)	364 (72.7%)	412.7% (775.9%)
Intra-hospital stillbirth rate per 1000 newborns in the hospital (proportion of total provincial stillbirths)	58.2 (29.3%)	37.8 (18.5%)	53.2 (22.0%)	60.5 (27.5%)	3.9% (−6.1%)

^a^a caesarean section was considered inevitable in case of lack of progress after artificial rupture of membranes and augmentation with oxytocin, repeated failed induction of labour with misoprostol, failed attempt at vacuum extraction, two or more previous caesarean sections, placental abruption, non-cephalic presentation with present foetal heartbeat, ruptured uterus, placenta praevia, or cord prolapse with present foetal heartbeat, as documented in the clinical files

**Fig. 2:**
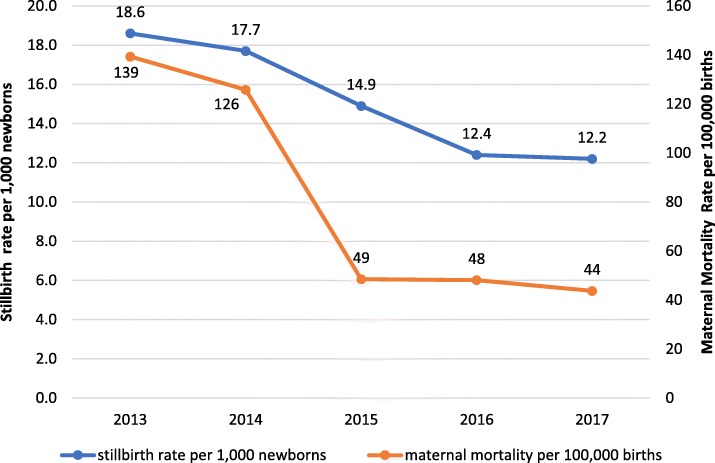
Institutional maternal mortality and stillbirth rates, 2013–2017, Tete Province

**Fig. 3:**
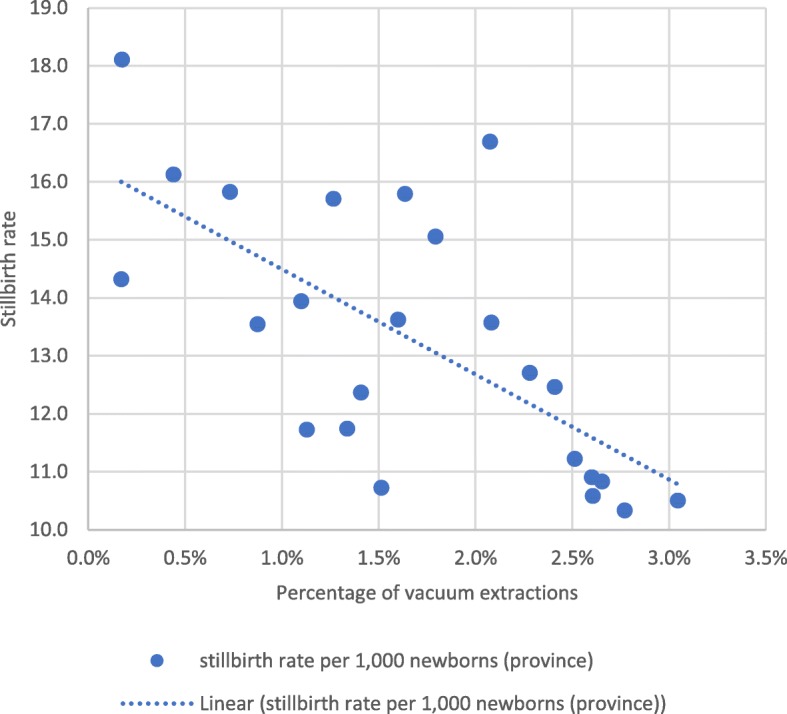
Association between the monthly percentage of vacuum extractions in institutional births versus institutional stillbirth rates in Tete province, 2015–2016

**Fig. 4:**
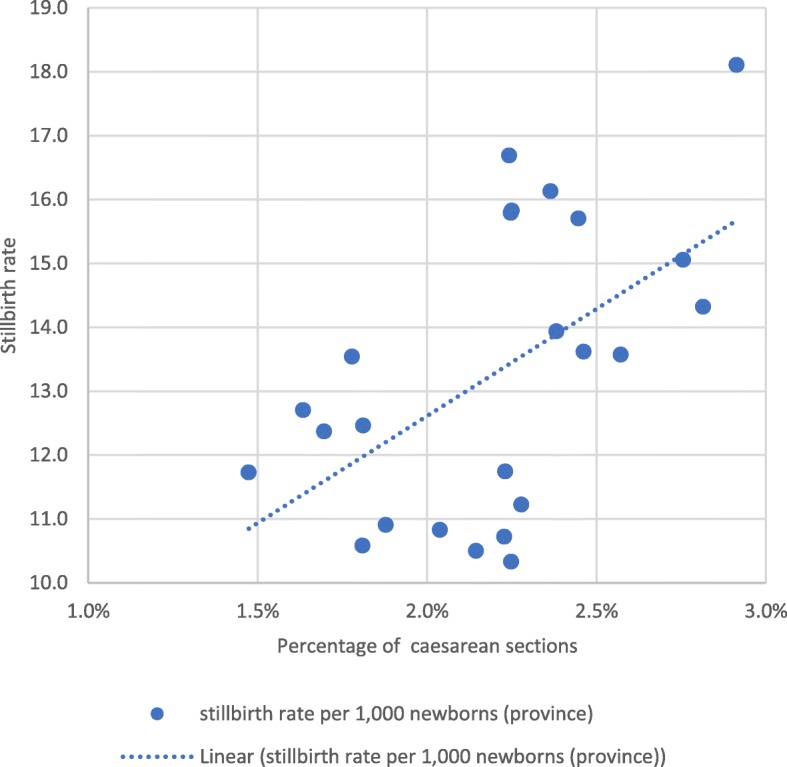
Association between the monthly percentage of caesarean sections in institutional births versus institutional stillbirth rates in Tete province, 2015–2016

At provincial level, maternal and perinatal outcomes after institutional childbirth improved between 2014 and 2017. Institutional maternal mortality per 100,000 births and stillbirths per 1000 newborns reduced considerably (Fig. 2). Case fatality rates of direct obstetric complications reduced from 2.2% in 2014 to 0.4% at the end of 2016 and 0.8% during 2017. The reduction in case fatality rates was similar in each direct complication, prolonged labour, obstetric haemorrhage, (pre-) eclampsia, and sepsis; in 2016, all achieved rates below 1% due to a combination of fewer maternal deaths and more frequent diagnosis and treatment of cases with complications. However, during 2017 the case fatality rates for (pre-) eclampsia and sepsis rose again to well above 1%. The monthly institutional stillbirth rate was inversely associated with the percentage of vacuum extractions among institutional births, but positively associated with the percentage of caesarean sections (Figs. 3 and 4).

Mozambique does not collect reliable routine data on neonatal deaths. Yearly admissions due to asphyxia to the neonatal intensive care unit in the provincial hospital fluctuated around 400–450 between 2014 and 2016, and the case fatality rate ranged from 21% in 2014 to 16% in 2015 and 29% in 2016. Nevertheless, the impression of the provincial paediatrician as well as of provincial programme managers is that perinatal outcomes in general, both stillbirth and neonatal death, had improved in recent years. Taken together with the increasing coverage of institutional births, it is likely that the improvements in institutional maternal and perinatal outcomes extend to population level.

### Enabling factors for implementation

It has been well documented that many interventions to improve health have been scientifically proven effective, but are not generally applied in daily practice, as was the case with Mozambique’s obstetric care guidelines [49]. The lack of implementation may be attributed to barriers at health system level, such as insufficient funding or human resources, or at health facility level, such as a lack of equipment or of capable midwives, a lack of leadership to ensure quality of clinical care or inadequate teamwork [50–52]. Rather than documenting barriers for implementation, this study chooses to present enabling factors, organised within an implementation science perspective, in factors related to a) the innovation itself, b) the structural and organisational environments in which the innovation is introduced, c) the people involved in the implementation (managers, staff, patients), and d) the process through which the implementation was rolled-out (adapted from Damschroder et al., 2009 and Chaudoir et al., 2013 [29, 30]).

- The innovation itself, vacuum extraction

An important key to success is the choice of the correct intervention for the problem which needs solving. In this case, the main problem to be addressed was the inadequate management of prolonged labour as principal cause of institutional maternal death and stillbirth. Vacuum extraction was re-introduced within a generally improved management of labour, including completion of the partogram and its use for timely decision-making, conservative interventions such as artificial rupture of membranes and augmentation with oxytocin intravenously, with caesarean section as last recourse. This course of action is in accordance with national and international guidelines, and was recommended by a senior expert with longstanding experience in Mozambique and good connections with several high-level personnel of the Ministry of Health, which made the advice unreservedly acceptable to Tete’s health personnel and managers.

Other positive aspects of vacuum extraction are directly related to the instrument. It is safe even in less-experienced hands, it offers the opportunity to regularly practice in non-urgent situations, for example in tired nulliparous women with a slight delay in the second stage of labour. It is a procedure of little complexity, which can be guided by few simple criteria and rules. The components of the instrument may be put together in a variety of combinations, which makes functional availability at peripheral level more likely. The positive effect of its successful use is immediate and rewarding for the practitioner (and the patient). This offers a more favourable option in the periphery compared with referral and summoning an ambulance or facing the possibility of an asphyxiated newborn or intra-partum stillbirth. The new handheld model vacuum extractor, received from national level, is small and easy to use, which encouraged its utilisation. Even after the model tended to break down with repeated use, MCH nurses were already convinced that vacuum extraction was an intervention they could perform and was beneficial to them and their patients. This meant they would look for, assemble, and use alternative equipment, which was already available to some extent in the province. Many additional resources were thus not required: checking inventories and subsequent redistribution was largely sufficient. Some experience with vacuum extraction was already present among the MCH nurses, which facilitated in-service training within existing teams. Similarly, some experience with augmentation with oxytocin or misoprostol, vacuum extraction, craniotomy and other destructive procedures, existed among obstetricians in the provincial hospital.

b) The structural and organisational environments in which the innovation was introduced

The Mozambican government and health sector are structures with a strong hierarchy, which consider the reduction of maternal mortality an important priority. Every month, data on maternal deaths must be presented directly from the provincial health directorate to the provincial governor and to the minister of health, which creates considerable pressure to find ways to success. This pressure is then translated down the chain of command, via province and districts to health facilities and individual staff, creating an environment where the reduction of maternal deaths must be on everybody’s agenda. It also sets the stage for a certain competition, for peer pressure between staff, health facilities, districts, and even provinces, to find answers and produce positive results, in the expectation to be recognised and rewarded for them. In a similar fashion, this hierarchical pressure also stimulated Tete’s provincial hospital to try harder to prove its status as tertiary referral hospital with a ‘model maternity’, especially after peripheral health facilities took the lead in improving emergency obstetric care and vacuum extraction.

Based on local research and evaluations, a common understanding had been created within the provincial health directorate on the main contributing factors towards the lack of progress in maternal and perinatal mortality, through which ‘inconvenient truths’ such as poor quality of care within health facilities could be assumed and subsequently addressed. The concurrent programme aimed at health system strengthening focussed on results-based management with evidence-based decision-making, using a ‘complex adaptive systems’ perspective. This led to improvements in health system management, governance, financial management, logistics and transport, human resources management and monitoring and evaluation throughout the provincial health sector. The process involved attention for stronger collaboration, promotion of transformational leadership and iterative learning. Taken together, this permitted the emergence of ‘champions of vacuum extraction’ at peripheral health facilities, whose positive experiences and good initial results aided in a gradually expanding practice of vacuum extraction at provincial, district and health facility level.

c) The people involved in the implementation

*Staff*: Many MCH nurses were afraid to perform a vacuum extraction, worried about possible damage to mother or child and of repercussions in case of failure to save the baby. Once the process of vacuum extraction and its components were demystified through simple rules and multiple possible assemblies, and once regular performance of vacuum extraction was presented as a routine professional practice of any competent MCH nurse, more staff were gradually willing to try out the procedure in practice. Any attempt to perform vacuum extraction was always praised, even when unsuccessful, because trying and failing was considered better than not trying at all. Many MCH nurses discovered quickly that vacuum extraction is not a difficult procedure, that it is largely safe for mother and child and that it forms a powerful tool for any MCH nurse to resolve difficulties in the second stage of labour without need to obtain outside assistance, avoiding unnecessary referrals or caesarean sections. In peripheral health facilities, MCH nurses realised that it was often easier for them to perform vacuum extraction than to convince the patient and her family that referral was necessary and to subsequently arrange an ambulance. The following quote from a group of MCH nurses in a provincial meeting on maternal and perinatal mortality summarises this process of empowerment:

*“We knew that a vacuum extraction is an instrumental childbirth which might reduce perinatal mortality and also reduce the anxiety of the mother in a situation of prolonged labour. But in the beginning, we were afraid and apprehensive. We needed individual change, to gain courage and start performing vacuum extractions when we as MCH nurses considered it necessary to save a foetus in danger of perinatal mortality. Now, when we do a vacuum extraction, it means a victory and an achievement to us.”*

In the provincial hospital, staff gradually gained the confidence to promote vaginal births rather than quickly deciding on a caesarean section. The understanding emerged that caesarean section is not always the best solution, that a vaginal birth may be safer for mother and baby, and that caesarean section might be best seen as a last resort if all else fails. Better collaboration within the multidisciplinary team, with clearer divisions of roles, more dialogue and second opinions aided in this process.

*Patients:* Although direct contributions from patients were not available, staff reports suggested that assistance with vacuum extraction during prolonged labour was gratefully received by patients, especially when it saved the baby and avoided referral for caesarean section in a far-away hospital. For socio-cultural reasons, vaginal births are traditionally better accepted than caesarean sections among Tete’s population. Referral to a health facility with surgical capacity is not popular, due to difficult travel, return transport costs for the patient and her family, fear of surgical intervention, and the unfortunate fact that often the baby cannot be saved even through surgery due to unavoidable delays related to long distances and poor transport infrastructure. Consequently, many women in Tete’s rural communities know sad histories of acquaintances or family members who experienced difficulties when giving birth, were referred, had a caesarean section but lost their baby and never got pregnant again. Vacuum extraction appears thus as a potentially acceptable tool to promote vaginal birth resulting in a live, healthy mother and baby at peripheral health facilities.

d) The process through which implementation was rolled-out

While a common understanding of the problem of prolonged labour and its possible solutions existed in the province, planning of the four interventions was very limited. Only the training was formally planned, the other interventions were suggested during the training or were included spontaneously shortly afterwards. The roll-out of the innovation did not follow the usual process in the Mozambican health system, consisting in widespread formal training of all staff involved with elaborate written guidelines and protocols. Rather, the implementation process in Tete was flexible and responsive to changes and resistance, relying mostly on personal initiatives of MCH nurses and other maternity staff, guided by few simple rules and criteria within their day-to-day routines, supervised through existing hierarchies by district medical officers and health directors. By largely avoiding classroom teaching, the impression that vacuum extraction is a difficult procedure which must be formally learned was avoided. This also prevented passive resistance from MCH nurses who had not benefitted from formal training and its accompanying per diem, which tends to be widespread in Mozambique [53].

Most encouragement was directed towards those maternities attending the largest numbers of institutional childbirths, ensuring enough cases to permit regular practice to gain experience and develop skills. Improved practices in these maternities also ensured an impact on a considerable number of childbirths, enough to provoke an impact at provincial level. Early positive results stimulated MCH nurses and district doctors to continue and expand the practice, creating a snowball effect, increasing practice and increasing benefits for maternal and perinatal outcomes, resulting in the creation of a virtuous circle.

Initial results were presented to the provincial government and the Ministry of Health, which created a pressure to move forward, as permitting a resurgence of maternal and perinatal mortality after its reduction was shown possible would mean a severe loss of face for Tete’s provincial managers. The practice of vacuum extraction was therefore included in the list of priority activities on which monthly feedback was required via phone message from districts directly to the provincial chief medical officer. Pressure for accountability on results via email, WhatsApp, phone messages, reports, meetings, etc. was strong and mostly public among provincial and district staff, as were prizes and praise for those performing well. As such, peer pressure aided in the establishment of a new cultural norm in which vacuum extraction is now considered a normal part of day-to-day practices of any competent MCH nurse or district doctor. However, this peer pressure was interrupted in 2017 due to staff changes at provincial level affecting the chief medical officer, chief public health officer, MCH manager and locally-based international technical advisor, which explains the partial relapse noted during 2017, especially in the implementation of all signal interventions as required for accreditation. Determined to maintain previous advances, steps to resume the regular accreditation and feedback system have been taken by relevant managers of the Provincial Health Directorate at the start of 2018.

## Discussion

This experience of Tete Province, Mozambique, with improvement of emergency obstetric care including vacuum extraction, shows that it is possible to make progress in maternal and perinatal mortality, even in a severely resource-constrained setting and without a large budget for interventions. The role of skilled and empowered MCH nurses and midwives appears as a crucial element, as described in other publications [54–56]. Once they are convinced of the benefits of vacuum extraction for themselves and for their patients, and once they are permitted and enabled to use this instrument as trusted professionals, they will take the chance with both hands and deliver results for mother and child. This study demonstrates Freedman’s statement in The Lancet: “The true engine of change in maternal health will not be the formal clinical guidelines, polished training curricula, model laws, or patient rights charters we produce. The engine will be the determination of people at the front-lines of health systems—patients, providers, and managers—to find or take the power to transform their own lived reality” [57]. Such transformation can be achieved with existing, proven interventions, when implemented with careful attention to the lessons from implementation science and systems thinking. Context and process of implementation are of crucial importance for success, although these tend to receive little attention in more traditionally designed programmes. The construction of a common goal through local learning requires extensive pre-intervention groundwork, sometimes over years, to ensure that a truly common understanding may be reached: the conviction that a problem exists which requires evidence-based solutions and that such solutions are within reach, despite all existing constraints. Promoting behaviour change in overworked health staff and managers facing many competing priorities requires a flexible approach, as it is impossible for top-down prescriptions to foresee and address all complexities of their day-to-day practices and experiences. Simple rules and appropriate incentives stimulate innovation, while dictated commands and attempts at control stifle local initiative and personal development. Rather than attempting to engineer change, one can create and then nurture the conditions for change to emerge [31, 58, 59].

The underutilisation of vacuum extraction has been repeatedly pointed out as a missed opportunity to improve emergency obstetric care in middle and low-income countries (most recently by Bailey et al., 2017 [15]), but interventions aiming to reverse this are still rare. Nolens et al., 2016 [28] described a successful intervention in the national referral hospital of Uganda, where vacuum extractions increased while caesarean sections remained stable and an association with improved maternal and perinatal outcome was strongly suggested. Others tended to present a more general focus on improvement of emergency obstetric care without specific mention of vacuum extraction [22–27, 60, 61]. Often an increase of caesarean section is described as a positive effect in such studies, but in view of Tete’s experiences and the emergence of many caesarean sections performed “too much, too soon”, this might need refinement in future work to separate unavoidable caesarean sections from those with less firm indications. In addition, future studies might include attention to potential cost-savings of using vacuum extraction instead of caesarean section in resource-constraint health systems, which remained outside the scope of this study.

This study depended on routine data and lived experiences during day-to-day work, without any formal data collection process for scientific purposes. This implies necessarily a concern for data quality, although it is unlikely that inadequate quality would be responsible for positive changes in such a wide range of obstetric care indicators. Possibly data quality may have improved with the increased attention for maternity monthly reports. Internal routine data quality assessments in 2015 and 2016 based on a methodology developed by World Health Organisation showed that completeness, internal and external validity of institutional childbirth data per health facility were sufficient to make them fit for purpose. The analysis of lived experiences without a formal note-taking system presents the chance of recall mistakes. This weakness was partly addressed using routinely prepared documentation (WhatsApp messages, minutes of meetings, etc.) and through dialogue and verification of findings and interpretations with all actors involved. The implementation framework ensured in-depth analysis of all possible relevant aspects, which diminished the chance that some vital component might have been overlooked.

Unfortunately, it was not possible to include direct voices and perspectives from patients and further research will be required to confirm or disprove the views received indirectly via health staff. Despite these limitations, given the importance and relevance of the topic, this study still presents lessons and insights useful for future efforts aimed at improvement of emergency obstetric care and re-introduction of vacuum extraction in similar areas and countries.

## Conclusions

The lessons from implementation science and systems thinking can contribute to surprising results in the improvement of emergency obstetric care including use of vacuum extraction, even in a severely resource-constrained setting and without a large budget for interventions. The creation of conditions for real change, with empowerment of the staff and managers at the front-line of day-to-day practice in emergency obstetric care in Tete province should serve as an inspiration for others in similar conditions and circumstances. The underutilisation of vacuum extraction in middle- and low-income countries is indeed a missed opportunity. Its reversion is possible even in resource-constrained settings and provides a good chance to make considerable difference to maternal and perinatal outcomes.
